# A Unique Core–Shell Structured, Glycol Chitosan-Based Nanoparticle Achieves Cancer-Selective Gene Delivery with Reduced Off-Target Effects

**DOI:** 10.3390/pharmaceutics14020373

**Published:** 2022-02-07

**Authors:** Bei Cheng, Hye-Hyun Ahn, Hwanhee Nam, Zirui Jiang, Feng J. Gao, Il Minn, Martin G. Pomper

**Affiliations:** 1 Department of Radiology, Johns Hopkins University School of Medicine, Baltimore, MD 21205, USA; bcheng6@jhu.edu (B.C.); hahn11@jhmi.edu (H.-H.A.); hnam4@jhmi.edu (H.N.); zirui.jiang@jhmi.edu (Z.J.); 2 Institute for NanoBioTechnology (INBT), Johns Hopkins University, Baltimore, MD 21218, USA; 3 Department of Physiology, Johns Hopkins University School of Medicine, Baltimore, MD 21205, USA; fgao9@jhmi.edu

**Keywords:** molecular-genetic imaging, reporter–probe pair, gene delivery, systemic delivery, toxicity

## Abstract

The inherent instability of nucleic acids within serum and the tumor microenvironment necessitates a suitable vehicle for non-viral gene delivery to malignant lesions. A specificity-conferring mechanism is also often needed to mitigate off-target toxicity. In the present study, we report a stable and efficient redox-sensitive nanoparticle system with a unique core–shell structure as a DNA carrier for cancer theranostics. Thiolated polyethylenimine (PEI-SH) is complexed with DNA through electrostatic interactions to form the core, and glycol chitosan-modified with succinimidyl 3-(2-pyridyldithio)propionate (GCS-PDP) is grafted on the surface through a thiolate-disulfide interchange reaction to form the shell. The resulting nanoparticles, GCS-PDP/PEI-SH/DNA nanoparticles (GNPs), exhibit high colloid stability in a simulated physiological environment and redox-responsive DNA release. GNPs not only show a high and redox-responsive cellular uptake, high transfection efficiency, and low cytotoxicity in vitro, but also exhibit selective tumor targeting, with minimal toxicity, in vivo, upon systemic administration. Such a performance positions GNPs as viable candidates for molecular-genetic imaging and theranostic applications.

## 1. Introduction

Despite recent advances in management, cancer remains the second leading cause of death globally and in the United States [1,2,3]. Over 60% of patients with solid tumors die from metastases, suggesting that early detection coupled with new, advanced therapies would improve outcomes [4,5]. Imaging and therapy can be performed concurrently with theranostics, combining a therapeutic with a diagnostic (imaging) agent, an approach of increasing interest and visibility [6,7,8,9]. Conventional strategies to engage tumor cells with imaging or toxic warheads in vivo through cancer-specific markers, such as membrane receptors, metabolic enzymes, and structural protein targets, often provide a measure of undesired, off-target effects [7,10,11,12,13]. Molecular-genetic imaging and therapy using reporter–probe pairs have been pursued in various ways for over two decades [14,15,16,17]. An advantage of the molecular-genetic approach over other theranostics is the capacity to produce the imaging and/or therapeutic agent specifically within the cancer cell, in situ, as the promoter activates production of these agents only when in contact with malignant tissues. We applied tumor-selective promoters, such as the progression elevated gene-3 promoter (PEG-3) [15] and astrocyte elevated gene-1 (AEG-1) [18], for cancer imaging through systemic delivery using a linear polyethyleneimine (PEI) nanoparticle (NP) as the delivery vehicle in vivo. However, superior agents are needed.

Non-viral vectors have certain advantages over viral vectors for systemic gene delivery, such as the potential for lower immunogenicity [19,20], lower likelihood of tumorgenicity [21], less sequestration in liver and spleen [22,23,24], and straightforward, scalable manufacturing [25,26]. Polycations, such as polyethylenimine (PEI), chitosan, and poly-beta-amino esters (PBAE), are useful non-viral vectors to deliver nucleic acids and proteins [27,28,29,30,31]. PEI is considered the gold standard for in vitro transfection [32]. However, the in vivo application of PEI has been hindered due to low transfection efficiency and moderate toxicity, which may derive from its high charge density [33]. The first polymer-based siRNA systemic delivery system tested in humans, CALAA-01, reduced platelet counts and proved toxic to liver and kidney during clinical trials [34].

Intracellular stimuli-responsive nanoparticles (NPs) that are colloidally stable and capable of the controlled release of therapeutics within targeted cells may be well-positioned to address toxicity and low transfection efficiency issues [35,36]. Commonly used chemical–stimuli-responsive NPs are pH- or redox-responsive [37]. By utilizing pH variation within the intracellular environment, i.e., cytosol (pH 7.4), lysosomes (pH 4.5–5), and endosomes (pH 5.5–6), pH-responsive NPs are designed to release contents within specific organelles. [34] Compared with pH-responsive NPs, redox-responsive NPs may be more versatile due to the substantially greater redox differences between healthy and malignant tissues, as well as intracellular versus extracellular environments [38,39,40,41]. Glutathione (GSH), one of the main regulators of cellular redox status, is synthesized exclusively in the cytosol. The intracellular GSH level (2–10 mM) is much higher than the extracellular GSH level (~2–10 µM) [41]. Additionally, GSH levels are elevated in many cancers, such as breast, ovarian, head and neck, and lung cancer [42]. It is reported that the tissue level of GSH in breast tumors was 56.8 times higher than of the peritumoral tissues [43]. Accordingly, an effective redox-responsive NP system would enhance the cytosolic delivery of nucleic acid within targeted cells.

**Scheme 1:**
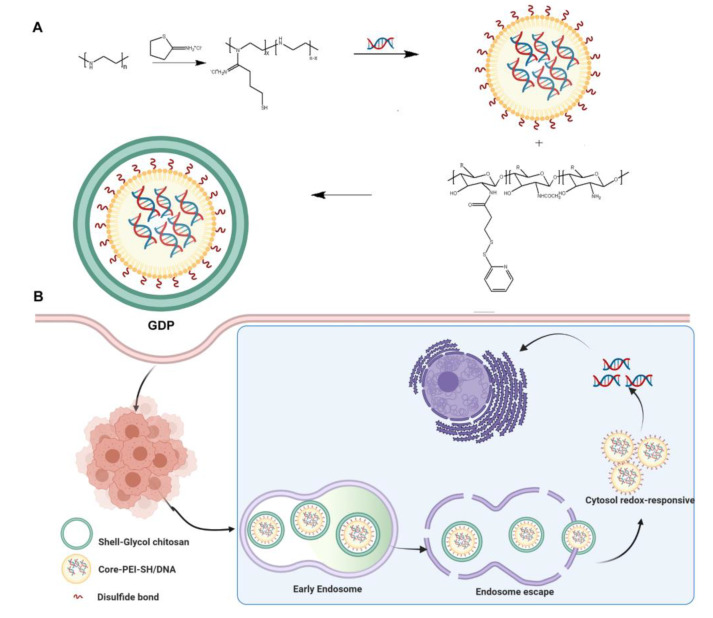
Schematic illustration showing the preparation (**A**) and cellular trafficking (**B**) of GNPs.

Building on our earlier work [44] with glycol chitosan (GC) NPs to deliver genetic material (siRNA), here we have formulated and screened a highly stable, redox-sensitive NP system, GCS-PDP/PEI-SH/DNA NPs (GNPs), for metastatic cancer theranostics. GNPs exhibit a unique core–shell structure formed between PEI-SH and DNA, and GCS modified with succinimidyl 3-(2-pyridyldithio)propionate (GCS-PDP) as the shell (Scheme 1). GNPs maintained colloidal stability and released DNA selectively within a highly reducing environment. GNPs showed high and redox-responsive cellular uptake, high transfection efficiency, and low cytotoxicity in vitro. They also exhibited superior tumor targeting with minimal toxicity in vivo. GNP is a promising translational nanocarrier for the systemic delivery of molecular-genetic theranostics.

## 2. Materials and Methods

### 2.1. Reagents and Cell Culture

Glycol chitosan, potassium persulfate, dithiothreitol (DTT), 2-iminothiolane (also known as Traut’s reagent), formaldehyde solution 36.5–38%, and 3-(4,5-dimethylthiazol-2-yl)-2,5-diphenyltetrazolium bromide (MTT) were purchased from Sigma-Aldrich (St Louis, MO, USA). l-Buthionine-sulfoximine was from Cayman Chemical (Ann Arbor, MI, USA). Linear PEI (PEI, molecular weight 25 kDa, transfection level) was obtained from Polysciences, Inc. (Warrington, PA, USA). Succinimidyl-3-(2-pyridyldithio)propionate (SPDP) and trehalose were purchased from TCI America (Portland, OR, USA). (5,5-Dithio-bis-(2-nitrobenzoic acid)) (DTNB) and nucleic acid dye YOYO-1 were obtained from Thermo Fisher Scientific (Waltham, MA, USA). CY5.5 NHS ester was obtained from Lumiprobe (Hunt Valley, MD, USA).

PC3 human metastatic prostate cancer cells were cultured in DMEM medium containing 10% fetal bovine serum (FBS) and 1% penicillin-streptomycin at 37 °C in a 5% CO_2_ atmosphere. The pSur-fLuc, in which firefly luciferase is driven by the survivin (Sur) promoter [45], was generated by replacing the mCMV enhancer and EF1 promoter in the pCpG free-*N*-mcs plasmid (Invivogen, San Diego, CA, USA) with the survivin promoter. The pCMV-fLuc [15], in which firefly luciferase is driven by the constitutive cytomegalovirus (CMV) promoter, was purified using QIAGEN Plasmid Midi Kit (Germantown, MD, USA). d-Luciferin was purchased from GoldBio (St Louis, MO, USA).

### 2.2. Polymer Synthesis

PEI was thiolated using Traut’s reagent. Briefly, PEI (20 mg, 0.8 µmol) was dissolved in 4 mL of 100 mM PBS at 80 °C containing 5 mM EDTA and was then degassed with nitrogen. After adding Traut’s reagent (1.65 mg, 12 µmol), the reaction mixture was stirred at room temperature (RT) for 2 h. The product was purified by dialysis using dialysis tubing with a molecular weight cutoff (MWCO) of 10 kDa (Repligen, Waltham, MA, USA) against deionized (DI) water containing 1 mM EDTA followed by DI water. The final product was aliquoted and stored at −80 °C before use. The degree of thiolation was quantified by DTNB assay (Thermo Fisher Scientific, MA, USA) according to a literature procedure [46].

GCS was depolymerized prior to use, according to a previous study [47]. Depolymerized GCS (40 mg, 3.1 µmol) was dissolved in 10 mL PBS (pH 8.0). SPDP (12.48 mg, 0.04 mmol) was dissolved in 1 mL DMSO, which was then added to the GCS solution. After stirring overnight at RT, the product was purified by dialysis using dialysis tubing with an MWCO of 3500 Da against DMSO followed by DI water. The GCS-PDP yield was quantified by measuring the UV absorbance of pyridine-2-thione at 343 nm. The GCS-PDP polymer was lyophilized and stored at −20 °C before use.

GCS-based disulfide-containing PEI derivative (GCS-ss-PEI) was used as the control polymer, which was prepared by mixing GCS-PDP with PEI-SH in TBS (10 mM, pH 7.0) overnight at RT. The product was purified by dialysis using dialysis tubing with an MWCO of 10 kDa against DI water.

### 2.3. Preparation and Characterization of GNPs

Plasmid DNA (0.2 mg/mL in nuclease-free water, 50 µL) was mixed with an equal volume of PEI-SH solution (0.2, 0.3 or 0.4 mg/mL) first, followed by addition of an equal volume of GCS-PDP solution (0.2 mg/mL). The mixture was incubated at RT for 2 h. The GNPs obtained were characterized according to the weight ratio of GCS-PDP to PEI-SH to DNA. For example, GNPs prepared using GCS-PDP at 0.2 mg/mL, PEI-SH at 0.4 mg/mL, and DNA at 0.2 mg/mL are referred to as a “1/2/1” formulation. The size and zeta potential of the GNPs were measured by dynamic light scattering (DLS) on a Zetasizer Nano ZS (Malvern Instruments, Malvern, UK). GNPs were diluted in TBS buffer (10 mM, pH 7.4) prior to measurement. The morphology of the NPs was characterized using transmission electron microscopy (TEM). Briefly, 10 µL GNP solution was placed on the formvar-coated grid and incubated for 15 min. After removing the GNP suspension, the grid was stained with 2% uranyl acetate for 30 sec and blotted with filter paper. The grid was air-dried and then mounted in Tecnai 12 BioTwin TEM (FEI, Hillsboro, Oregon) for imaging. Images were recorded by a Megaview III wide-angle camera (EMSIS GmbH, Germany).

### 2.4. Colloid Stability of GNPs

To determine the long-term stability of the GNPs in TBS, a solution of the GNPs (0.1 mg/mL) was incubated in a benchtop shaker at 100 rpm at 37 °C for one month (Benchmark Scientific Inc, Edison, NJ, USA). Changes in the particle size distribution were monitored by DLS. The stability of GNPs in a simulated physiologic environment was evaluated by incubating them with 10 mM TBS, 150 mM PBS, or DMEM complete medium plus 10% FBS for 15 min. The GNP size was measured by DLS, and the DNA release from GNPs was analyzed using a 0.8% agarose gel [48].

### 2.5. Polymer Characterization

The molecular weight and polydispersity index (PDI) of the polymers were determined by gel permeation chromatography (GPC) (Agilent 1260 Infinity II, Santa Clara, CA, USA), where PBS (pH 7.4) was the mobile phase with a flow rate of 0.5 mL/min. Polymer structures were analyzed by 1H NMR on a Bruker Avance 500 (Billerica, MA, USA) with deuterated water (D2O). A trace amount of acetic acid-d4 was added to solubilize PEI.

### 2.6. DNase Protection

To investigate DNA protective effects, naked DNA, PEI/DNA, and GNPs containing DNA were incubated with or without 0.2 µL DNase I (New England Biolabs, Ipswich, MA, USA) for 10 min at 37 °C. The samples were then incubated at 75 °C for 10 min to inactivate the DNase. To release the undigested DNA, all samples were incubated with heparin (120 µg/mL) and DTT (10 mM) for 2 h and then separated on a 0.8% agarose gel and visualized with the ChemiDoc MP Imaging System (Bio-Rad, Hercules, CA, USA).

### 2.7. Intracellular Distribution of GNPs

Plasmid DNA and GCS-PDP were labeled with nucleic acid dyes YOYO-1 and Cy5.5-NHS, respectively, which were then used to generate fluorescent GNPs. PC3 human prostate cancer cells were seeded on a coverslip at a density of 200,000 cells per well in a 12-well plate. Cells were allowed to grow overnight, and were then incubated with the fluorescent GNPs for 4 h. Cells were fixed with 4% formaldehyde. Cells were labeled with Hoechst (H3570, Thermo Fisher Scientific) and anti-actin antibody (A5441, Sigma-Aldrich), and were then imaged using a Zeiss LSM 780 confocal microscope (Carl Zeiss AG, Oberkochen, Germany).

To determine whether GNPs escaped from lysosomes and released DNA into the cytosol, plasmid DNA was labeled with YOYO-1 and was then used to generate fluorescent GNPs (2/2/1). PC3 cells were seeded in a 35 mm glass-bottom dish (MatTek, Ashland, MA, USA), grown overnight, and were then incubated with YOYO-1-labeled GNPs for 1 h. Cells were washed with PBS and then stained with Hoechst and Lysotracker Red DND-99 (L7528, Thermo Fisher Scientific), which were analyzed using an LSM 780 confocal microscope.

### 2.8. Cell Transfection

pCMV-fLuc was used to test in vitro transfection efficiency. PC3 cells were seeded in a 24-well plate at a density of 80,000 cells per well. After 24 h, the medium was replaced with 500 µL of fresh medium plus 100 µL of a solution of the GNPs with a plasmid concentration of 10 µg/mL. After incubation for 48 h, the medium was removed, and cells were washed with PBS. Luciferase activity was measured using a Luciferase Assay System (E1500, Promega, Madison, WI, USA) and was recorded by a SpectraMax i3X plate reader (Molecular Device, San Jose, CA, USA). The total protein concentration was measured using a Coomassie protein assay kit (23200, Thermo Fisher Scientific). jetPRIME^®^ and in vivo jetPEI (Polyplus-transfection SA, Illkirch-Graffenstaden, France), Lipofectamine 2000 (Invitrogen, Carlsbad, CA, USA), and 25 kDa PEI were used as controls.

### 2.9. Cytotoxicity

The MTT assay was used to assess cell viability according to a literature procedure [49]. Briefly, PC3 cells were seeded in a 96-well plate at a density of 20,000 per well. After 24 h, the medium was replaced with 100 µL of fresh medium and 20 µL of a solution containing GNPs with a plasmid concentration of 10 µg/mL. After 48 h, MTT solution (0.5 mg/mL) was added to each well and was incubated for 4 h at 37 °C. Absorbance at 595 nm was recorded. Cell viability (%) = (A595 nm for the treated cells − A595 nm for the blank without cells)/(A595 nm for the non-treated control cells − A595 nm for the blank without cells) × 100. The Annexin V-APC/7-AAD kit (Thermo Fisher) was used to measure cell apoptosis. PC3 cells were seeded in a 24-well plate at a density of 80,000 per well. After 24 h, the medium was replaced with 500 µL of fresh medium plus 100 µL of a solution of GNPs with a plasmid concentration of 10 µg/mL. After incubation for 48 h, cells were trypsinized and stained with Annexin V and 7-AAD. For each sample, 10,000 cells were analyzed by a BD AccuriTM C6 Plus flow cytometer (BD Biosciences, San Jose, CA, USA).

### 2.10. Biodistribution of GNPs

Procedures related to animal care and treatment were approved by the Johns Hopkins Animal Care and Use Committee, and met the guidelines of the National Institutes of Health Guide for the Care and Use of Laboratory Animals. A metastatic prostate cancer model was generated by intravenous (IV) injection (tail vein) with 1 × 106 PC3/ML cells [50] to NSG mice (NOD/Shi-scid/IL-2Rrnull, 4–6 weeks old). Mice were randomly divided into four groups (*n* = 3 per group): Group 1 for 1/1/1 GNPs, Group 2 for 1/1.5/1 GNPs, Group 3 for 1/2/1 GNPs, and Group 4 for in vivo jetPEI/DNA NPs (N/P = 6, prepared in 5% glucose, according to the manufacturer’s protocol). GNPs were lyophilized with trehalose and achieved a final concentration of 8% after reconstitution. Each mouse received an IV injection (tail vein) of 200 µL of GNPs containing 40 µg DNA. After 3 days, mice were anesthetized with 3% isoflurane and injected intraperitoneally (IP) with 100 µL of D-luciferin (30 mg/mL). Bioluminescence images (BLI) were taken 3 min after the D-luciferin injection using an in vivo imaging system (IVIS, PerkinElmer, Waltham, MA, USA). Mice were immediately euthanized by cervical dislocation under anesthesia, and organs were isolated for IVIS imaging. BLI levels were analyzed by Living IMAGE Software (PerkinElmer).

### 2.11. Toxicity of GNPs In Vivo

BALB/c (6–8 weeks old, Jackson Labs, Bar Harbor, ME, USA) and CD1 mice (8–10 weeks old, Charles River Laboratories, Wilmington, MA, USA) were used for in vivo toxicity studies. Mice were randomly divided into five groups (*n* = 5–7): Group 1 for 1/1/1 GNPs, Group 2 for 1/1.5/1 GNPs, Group 3 for 1/2/1 GNPs, Group 4 for in vivo JetPEI/DNA NPs, and Group 5 for those un-treated. DNA doses were 1.1 mg/kg for BALB/c mice and 1.5 mg/kg for CD1 mice. After 48 h, blood, liver, and lung were analyzed. Complete blood analyses, including counting of white blood cells (WBCs), red blood cells (RBCs), and platelets (PLTs), were performed using a Scil Vet abc hematology analyzer (Scil Animal Care Company, Barrie, ON, USA). Clinical chemistry parameters, including blood urea nitrogen (BUN), glucose (GLU), alkaline phosphatase (ALP), total protein (T-Pro), alanine aminotransferase (ALT), and creatinine (Cre) were measured using a Spotchem EZ chemistry analyzer (ARKRAY USA, Edina, MN, USA). Lung and liver were preserved in 10% formalin for 24 h, and then embedded in paraffin. The 4 µm sections were stained with hematoxylin and eosin (H&E) for histologic analysis.

### 2.12. Statistics

For each experiment, we provided the sample size, statistical tests, and the *p*-values in each figure or legend. Unless otherwise noted, data were expressed as mean ± SD (standard deviation). *p*-values < 0.05 were considered statistically significant.

## 3. Results

### 3.1. Preparation and Characterization of GNPs

For PEI–SH synthesis, the optimized feeding ratio, i.e., the ratio of PEI to Traut’s reagent, was 1:15, which achieved a modification yield of 44.7 ± 0.4%, indicating that 1 PEI chain was modified with an average of 6.7 thiol groups. Gel permeation chromatography (GPC) analysis showed that no unexpected crosslinking occurred in the PEI polymer during the thiol modification (Figure S1A). To enhance GCS solubility in aqueous medium [47], we first depolymerized the GCS from 242.2 kDa to 12.8 kDa (Figure S1B). The depolymerized GCS was further conjugated with SPDP, and the readout from UV–VIS spectroscopy indicated that the degree of modification of GCS-PDP was 68 ± 1.2 µmol SPDP groups per gram of GCS-PDP. 1HNMR of GCS-PDP showed pyridine proton chemical shifts at 7.32, 7.88, and 8.42 ppm (Figure S1C). We generated a library of 13 GNPs using the ratios of GCS-PDP to PEI and to DNA that varied from 0.1/2/1 to 4/2/1, and 2 redox-sensitive control NPs using the GC-ss-PEI copolymer (1HNMR spectra in Figure S4) to DNA at ratios of 2.5/1 and 3/1, respectively (Table 1). Unless otherwise noted, all DNA used for in vitro experiments was pCMV-fLuc [15]. The average particle size (mean ± SD) of GNPs ranged from 141.7 ± 1.3 to 228.8 ± 10.8 nm, and their polydispersity (PDI) was <0.2, indicating that the polymer can condense DNA to form GNPs within a narrow size distribution. Both DNA GNPs and GCS-ss-PEI/DNA had a much lower surface charge (~10.0–16.5 mV) than PEI/DNA NPs (22.9 mV) in TBS buffer (Table 1), indicating a partial consumption of secondary amines in PEI by conjugation with GCS, consistent with a previous study [51]. To determine whether GNPs could be lyophilized for long-term storage and for in vivo study, the GNPs were lyophilized with trehalose. The reconstituted GNPs showed no apparent hydrodynamic size change compared with fresh GNPs (Figure S2).

### 3.2. GNPs Maintain Colloid Stability

**Figure 1:**
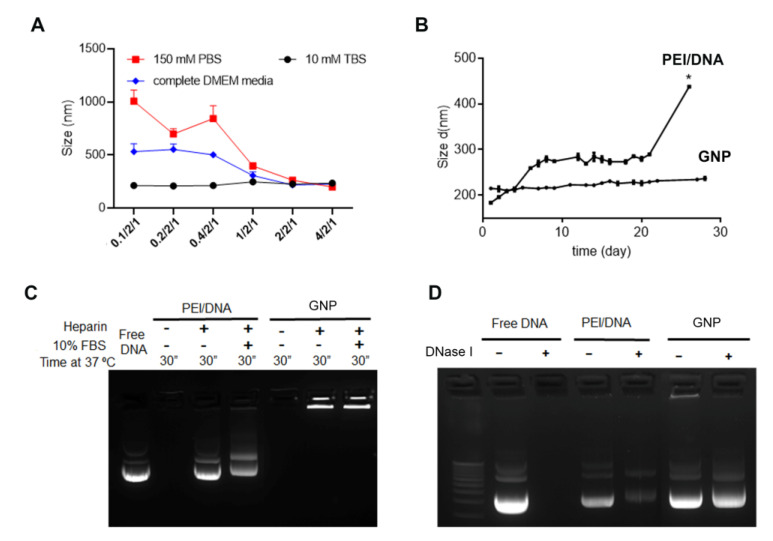
GCS-PDP/PEI-SH/DNA NP maintains stability under different conditions. (**A**) Hydrodynamic size changes of GNPs in different media; (**B**) hydrodynamic size change of PEI/DNA and GNPs in 10 mM TBS buffer at 37 ℃ over 30 days) (* indicates PEI/DNA complex aggregation, with polydispersity index = 0.806); (**C**) agarose gel electrophoresis assay of GNPs in the presence of heparin and 10% FBS; and (**D**) agarose gel electrophoresis assay of GNPs in the presence of DNase I. Each bar represents mean ± standard deviation (*n* = 3).

To a point, incremental increases in the GCS-PDP/DNA ratio improved the colloidal stability of GNPs in TBS buffer, PBS buffer, and DMEM (Figure 1A). DMEM supplemented with 10% FBS recapitulates human plasma with respect to the composition of amino acids, cations, and anions, but differs in the total protein amount [52]. The threshold ratio to maintain the colloidal stability of GNP in these buffers was 1/2/1, and any further increment in the GCS-PDP/DNA ratio did not significantly affect the size of the GNPs (Figure 1A). Compared with PEI/DNA NPs, GNPs had significantly higher long-term aqueous stability (Figure 1B). Moreover, in 10% FBS solution and anionic heparin solution, GNPs could protect from DNA loss better than PEI/DNA NPs (Figure 1C). In addition, DNA in the GNP was well protected from DNase I digestion (Figure 1D). In contrast with other nucleic acid delivery systems that incorporate cargo through electrostatic interactions and are inherently unstable [53], the GNPs proved stable to heparin displacement and serum, and showed reduced sensitivity towards nuclease digestion. Compared to PEI/DNA NPs, the enhanced stability of GNPs may be due to the shell structure formed by the PEI-SH and GCS-PDP linkage.

### 3.3. GNPs Release DNA Selectively in a High Reducing Environment

**Figure 2:**
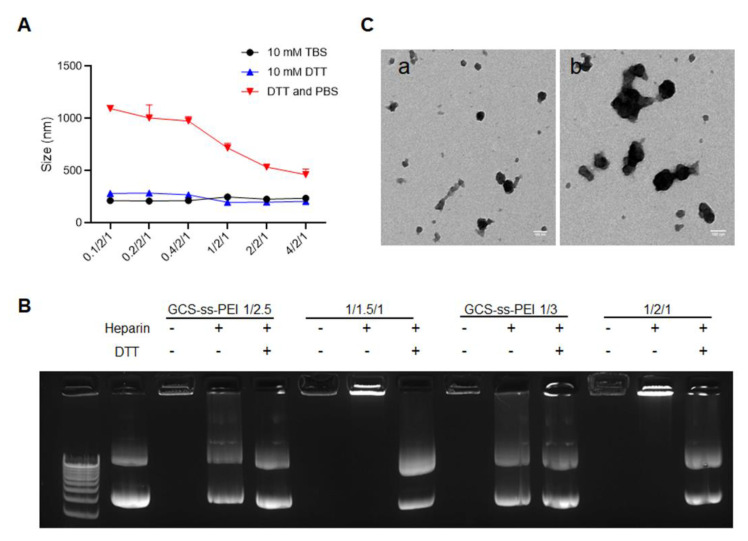
GNPs release DNA selectively in a high reducing environment. (**A**) The hydrodynamic size changes of GNPs in media of high redox potential. The ordinate represents the weight ratio of GCS-PDP to PEI-SH to DNA. Each bar represents mean ± standard deviation (*n* = 3); (**B**) agarose gel electrophoresis assay of GNPs and GCS-ss-PEI/DNA NPs in the presence of DTT and heparin; and (**C**) typical TEM images of GNPs before (a) and after (b) co-incubation with DTT and heparin (scale bar =100 nm).

We evaluated the redox sensitivity of GNPs by using DTT to simulate the reducing intracellular environment of cytosol and cancer cells [54,55,56,57,58]. DTT alone did not affect GNP size, while the co-treatment of DTT and PBS increased the size of GNPs, and the size increment was negatively correlated with the GCS-PDP level (Figure 2A). The redox sensitivity of GNPs was further evaluated by agarose gel electrophoresis (Figure 2B). Heparin alone did not induce DNA release from the GNPs, but it induced DNA release from PEI-DNA or GCS-ss-PEI DNA NPs (Figure 2B and Figure S3). DNA was released from GNPs when treated with both DTT and heparin, and the DNA release occurred in a heparin dose-dependent manner. The TEM of GNPs (1/1.5/1) with or without DTT and heparin co-treatment demonstrated that redox stimuli could induce a rapid degradation from the spherical GNPs into irregularly shaped and enlarged structures (Figure 2C).

These results suggest that GNPs not only has an enhanced stability over the NPs prepared by condensing DNA through electrostatic interactions, such as PEI-DNA, but are also capable of releasing DNA under high reducing conditions selectively. Due to the electrostatic interaction and redox-reactive core–shell structure, the new GNPs tested as stable in an environment with physiologic ionic strength and low redox potential, i.e., blood circulation, while they release DNA under conditions of physiologic ionic strength and high redox potential, i.e., the intracellular environment of a cancer cell. The redox potential of GSH/oxidized GSH in the cytoplasm is −240 mV, which is 110 mV more reduced than in extracellular space [59]. The high reducing environment within the cytosol facilitates the site-specific release of nucleic acids by GNPs.

### 3.4. GNPs Demonstrate High, Redox-Responsive Cellular Uptake

**Figure 3:**
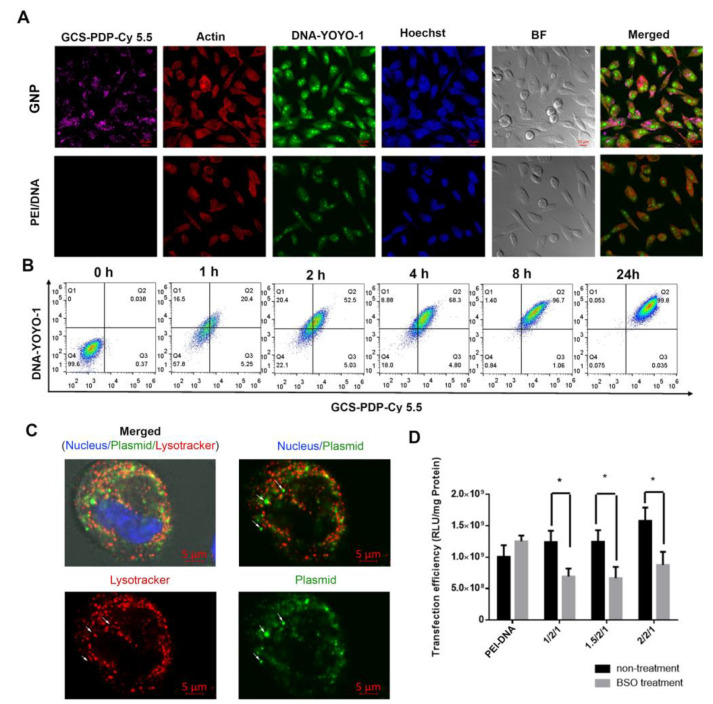
Uptake of GNP (1/1/1) in PC3 cells. (**A**) Confocal microscopy indicates the cellular distribution of GNPs and PEI/DNA NPs in PC3 cells after 4 h of incubation. GCS-PDP was labeled with Cy5.5 and DNA was labeled with YOYO-1; (**B**) FACS indicates the uptake of GNPs in PC3 cells at different time points; and (**C**) confocal images of PC3 cells after co-incubation with GNPs for 1 h. Small white arrows point to plasmids that are not in the lysotracker-positive compartment (lysosomes), and (**D**) the effect of intracellular GSH level on the transfection efficacy of redox-sensitive nanoparticles (data are analyzed by unpaired Student’s *t* test, * *p* < 0.05).

As cellular uptake is the initial process that must occur for the GNP to deliver its cargo effectively, we dually labeled GNPs by conjugating DNA with YOYO-1 (green) and GCS-PDP with Cy 5.5 (near-infrared) and performed real-time analysis of NP cellular uptake in PC3 cells. Compared to PEI/DNA (1/1) NPs, GNPs (1/1/1) showed higher YOYO-1-labeled DNA uptake levels after 4 h of incubation (Figure 3A). The flow cytometry showed that the cellular uptake level of GNP (1/1/1) increased over time and reached completion by ~8 h (Figure 3B).

Since it contains the low-molecular-weight redox component GSH, cytosol is a highly reducing environment with a GSSG/GSH ratio of ~1/1300 [60,61,62], providing an ideal location for GNPs to release their contents. To test if GNPs (1/1/1) could escape from the late endosomes/lysosomes, YOYO-1-labeled GNPs were first incubated with PC3 cells, followed by incubation with Lysotracker Red DND-99. The results show that some of the YOYO-1-labeled DNA had escapes from the lysotracker-positive compartment (Figure 3C), suggesting that the GNPs release DNA into the cytosol. To test if the cytosol DNA released from GNPs depended on the GSH level, we treated PC3 cells with a cellular GSH depletion agent, buthionine sulphoximine (BSO) [63,64] during transfection. We found that three GNP formulations (1/2/1, 1.5/2/1, and 2/2/1) showed a significant decrease in transfection efficiency in response to BSO treatment (Figure 3D). Together, these data demonstrate that GNPs show high, redox-responsive cellular uptake in PC3 prostate cancer cells. As intratumoral GSH levels are higher than those of non-malignant tissue, and more than three hundred times higher than plasma [65,66,67], the (reducible) disulfide bonds in the core–shell structure of GNPs undergo an intracellular reduction owing to the high concentration of GSH in the cytosol of the PC3 cells.

### 3.5. GNPs Exhibit High Transfection Efficiency with Low Cytotoxicity

**Figure 4:**
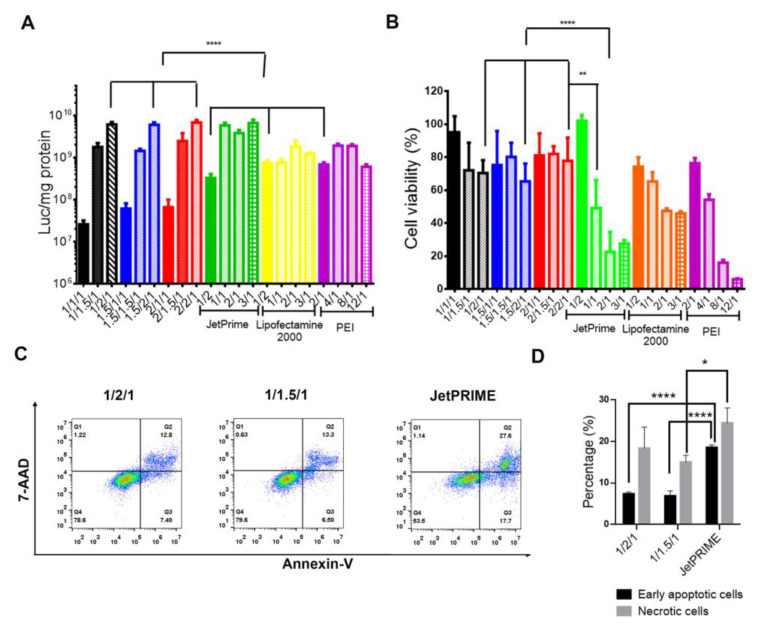
Optimization of GNPs to achieve high transfection efficiency and low cytotoxicity in PC3 cells. (**A**) In vitro transfection efficiency of different formulations measured by bioluminescence; (**B**) cytotoxicity of different formulations as determined by MTT assay; and (**C**,**D**) the percentage of early apoptotic and necrotic cells after treatment with GNPs or PEI/DNA NPs was quantified by flow cytometry. Each bar represents mean ± standard deviation. All data are analyzed by one-way ANOVA and Tukey’s multiple comparisons test (* *p* < 0.05, ** *p* < 0.01, **** *p* < 0.0001).

To screen for GNPs with high transfection efficiency and low cytotoxicity, nine different NPs containing CMV-fLuc plasmid were tested in PC3 cells (Figure 4A), where the transfection efficiency was quantified by luciferase assay. The results showed that the PEI-SH level was positively correlated with transfection efficiency, and that three formulations, 1/2/1, 1.5/2/1, and 2/2/1, exhibited a high transfection efficiency, which was comparable to jetPRIME and superior to Lipofectamine 2000 and the PEI 25K (Figure 4A). However, analysis for cytotoxicity showed that cell viability in the GNP group was significantly higher than that for jetPRIME (Figure 4B), and the differences were more dramatic in high FBS medium (Figure S5). Similarly, based on the annexin V/7-AAD assay, which differentiates intact cells (Annexin V-negative and 7-AAD-negative), early apoptotic cells (Annexin V-positive and 7-AAD-negative), and late apoptotic or necrotic cells (Annexin V-positive and 7-AAD-positive), GNPs (1/1.5/1 and 1/2/1) had a significantly lower number of apoptotic cells than jetPRIME (Figure 4C,D). The average percentage of early apoptotic cells were 7.4% in 1/2/1 GNP, 6.9% in 1/1.5/1 GNP, and 18.6% in jetPRIME. The average percentage of late apoptotic or necrotic cells were 18.4% in 1/2/1 GNP, 15.1% in 1/1.5/1 GNP, and 24.5% in jetPRIME. Those results indicate that GNPs have high transfection efficiency in vitro and, importantly, show significantly lower cytotoxicity than commercially available transfection reagents, including jetPRIME and Lipofectamine 2000. Even though the 1/1/1 GNPs show a relatively low transfection efficiency, the delicate balance between high transfection efficiency and low toxicity encouraged us to choose 1/1/1, 1/1.5/1, and 1/2/1 GNPs for in vivo animal tests.

### 3.6. GNPs Show Superior Tumor Targeting with Minimal Toxicity In Vivo

**Figure 5:**
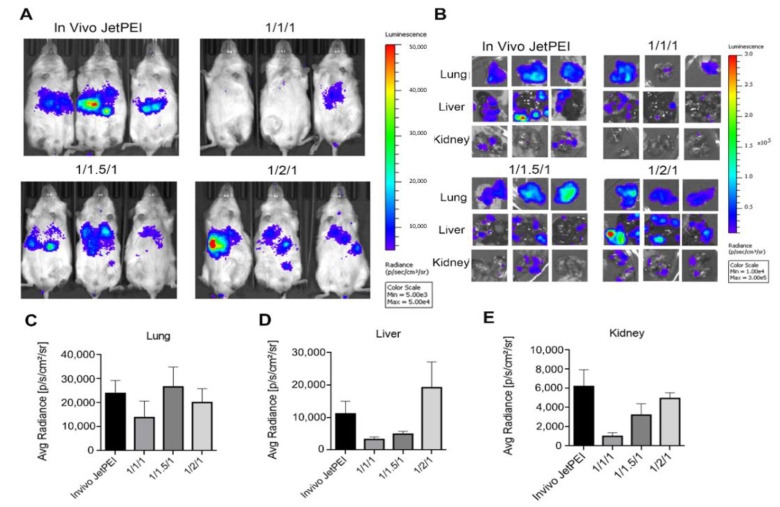
Transgene expression of different formulations in an experimental model of metastatic prostate cancer. (**A**) In vivo BLI of PC3-ML tumor-bearing mice after treatment with GNP and in vivo jetPEI/DNA NPs for 72 h; (**B**) ex vivo BLI images of in vivo jetPEI, 1/1/1, 1/1.5/1 and 1/2/1 GNPs treated mice; and (**C**–**E**) summary of luciferase expression level in the lung (**C**), liver (**D**), and kidney (**E**) at 72 h (*n* = 3–5 mice per group).

To evaluate the in vivo tumor targeting performance, three GNPs (1/1/1, 1/1.5/1, and 1/2/1) were tested in the PC3/ML mouse model [18] of human metastatic prostate cancer. We delivered pSur-fLuc DNA systemically, in which firefly luciferase was driven by the survivin (Sur) promoter, which can specifically induce transgene expression in cancer cells [68,69,70,71]. We first tested GNPs (1/2/1) and in vivo jetPEI containing pSur-fLuc as test DNA, in healthy NSG mice. Little-to-no luciferase expression in non-malignant tissues resulted (Figure S6). After confirming that the PC3 xenograft mouse model mainly developed tumors in the lung and liver [50,72], we administered three GNPs and in vivo jetPEI to these mice. Similar to transfection efficiency in vitro, the 1/1/1 GNP group showed the lowest BLI signal (Figure 5A). Compared with in vivo jetPEI, the 1/2/1 GNP group demonstrated a similar BLI signal in the lung but slightly higher signal in the liver, where most metastatic deposits were identified (Figure 5B–E).

**Figure 6:**
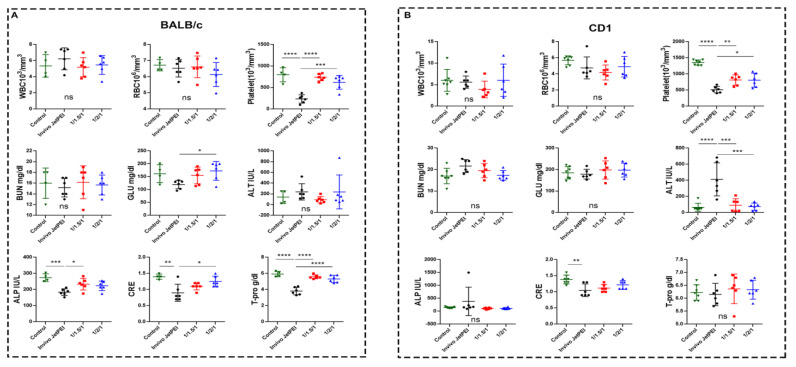
Complete blood analyses and clinical chemistry parameters of BALB/c (**A**) and CD-1 (**B**) mice treated with in vivo JetPEI or NPs (1/1.5/1 and 1/2/1). White blood cell (WBC), red blood cell (RBC), platelet (PLT), blood urea nitrogen (BUN), glucose (GLU), alkaline phosphatase (ALP), total protein (T-Pro), alanine aminotransferase (ALT), and creatinine (Cre) were measured (*n* = 5–7 mice per group; and data were analyzed by one-way ANOVA; ns, no significant difference, * *p* < 0.05, ** *p* < 0.01, *** *p* < 0.001, and **** *p* < 0.0001).

CD-1 is a classic outbred strain for toxicology studies, while BALB/c mice are sensitive to acute chemical injury and liver disease that may provide additional information about toxicity [73]. To test toxicity in vivo, both BALB/c and CD-1 mice received two GNPs (1/1.5/1 and 1/2/1) or in vivo jetPEI. After 48 h, complete blood analyses (white blood cell (WBC), red blood cell (RBC), and platelet (PLT) count) and clinical chemistry parameters (blood urea nitrogen (BUN), glucose (GLU), alkaline phosphatase (ALP), total protein (T-Pro), alanine aminotransferase (ALT), and creatinine (Cre)) were measured (Figure 6), followed by the histopathologic analysis of the liver and lung. The most consistent toxicity identified was a reduction in PLT counts in the BALB/c mice compared with their untreated counterparts, with a 70% reduction in the in vivo jetPEI group. Our GNPs showed a significantly lower decrease in the PLT counts than did animals in the in vivo jetPEI group, namely, for the 1/1.5/1 GNP group (*p* < 0.0001) and the 1/2/1 GNP group (*p* < 0.001) (Figure 6A). Compared with the untreated, control CD-1 group, there was a 62% reduction in the PLT counts for the in vivo jetPEI group, which was significantly lower than the reduction in the 1/1.5/1 GNP group (*p* < 0.01) and the 1/2/1 GNP group (*p* < 0.05) (Figure 6B). In the BALB/c cohort, other parameters that demonstrated higher toxicity for the in vivo jetPEI group than for the GNP groups included GLU, ALP, CRE, and T-Pro. For the CD-1 mice, ALT was also significantly higher in the in vivo jetPEI group than for both GNP groups.

**Figure 7:**
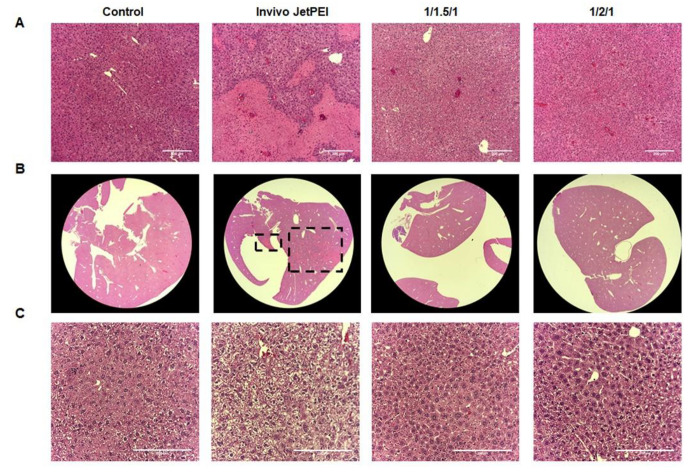
Histopathology of various NPs in BALB/c and CD1 mice. (**A**) H&E staining of liver from Balb/c mice treated with in vivo jetPEI/DNA NPs and GNPs (1/1.5/1, 1/2/1) under 10× (**A**) and 1.25× magnification (**B**); (**C**) H&E staining of liver from CD1 mice treated with in vivo jetPEI/DNA NPs and GNPs (1/1.5/1, 1/2/1) NPs under 20× magnification. Scale bar: 200 µm.

At histopathology, we found severe liver necrosis in the 5 out of 6 BALB/c mice (83%) treated with in vivo jetPEI, compared with only 1 case of mild liver necrosis from either the 1/1.5/1 or 1/2/1 GNP group (Figure 7). Those results demonstrate that GNPs, particularly the 1/2/1 GNP group, show not only superior tumor targeting efficiency, but also minimal toxicity in vivo in these preliminary studies.

## 4. Conclusions

NPs that carry nucleic acid can achieve disease-specific expression either through the composition of the NP or by tailoring the molecular payload itself. The rapid clinical deployment of cationic lipids for delivering SAR-CoV-19 mRNA during the pandemic underscores the utility of such reagents. Recent studies have employed polymer-based NPs, pH-sensitive copolymers, or targeting ligand conjugates, such as ethylamino-conjugated GC (chitosan) [74], poly(amidoamine)-conjugated CS [75], poly(lactide-*co*-glycolide)-graft-PEI [76], PEG-conjugated CS [77], and trimethyl CS-cysteine conjugates [78]. Despite the reasonable therapeutic effects, however, the efficiency of the cytosolic delivery of those agents needs further attention as accumulation in the cytosol is essential for theranostic applications. [79] The marked difference in redox environments between healthy and malignant tissues, and between the cytosol and extracellular space, can be exploited to facilitate cytosolic delivery of nucleic acids.

We searched for a biocompatible vehicle for systemic, tumor-selective delivery of genetic material. Our initial attempt utilized l-PEI due to its high transfection efficiency, leaving the specificity to activation of tumor cell-specific promoters. [80] The NP created herein has properties balanced between efficient cytosolic release of nucleic acid and colloid stability in vivo. The core–shell GNPs attained colloid stability through dense coating by GCS polymer brushes that provided steric stabilization when swelling in water [81]. Our results indicate that GNPs containing higher amounts of GC exhibit greater colloidal stability under physiologic conditions. To release nucleic acid in the cytosol, GNPs are initially internalized into cancer cells followed by endosomal escape through a proton-sponge effect. [82] They are eventually unpacked in the cytosol in response to the high reducing environment present within cancer cells. Our findings also show that the PEI-SH-to-DNA-weight ratio and the molecular weight of GCS-PDP had a significant impact on transfection efficiency. GNPs alleviate one of the most notorious side effects of certain commercial NPs, namely, platelet aggregation, induced by the instability of electrostatically complexed NPs.

In summary, GNPs can mitigate toxicity associated with PEI by reducing platelet aggregation and toxicity to liver. We have outfitted GNPs with a sensitive redox system that enhances transfection efficiency in vivo, through systemic administration, ultimately for theranostic applications.

## Figures and Tables

**Table 1:** Physicochemical properties of DNA NPs.

DNA NP Type	Ratio	Hydrodynamic Diameter ± SD, nm	PDI	ζ-Potential ± SD, mV
GNPs	0.1/2/1	143.1 ± 4.4	0.14 ± 0.01	16.5 ± 2.8
	0.2/2/1	142.6 ± 2.4	0.15 ± 0.02	16.5 ± 0.2
	1/2/1	165.6 ± 2.6	0.14 ± 0.03	14.3 ± 0.3
	2/2/1	226.6 ± 1.3	0.16 ± 0.02	14.3 ± 1.8
	4/2/1	228.8 ± 10.8	0.14 ± 0.04	16.7 ± 1.0
	1/1.5/1	151.3 ± 1.9	0.1 ± 0.03	14.0 ± 0.6
	1.5/1.5/1	144.0 ± 3.9	0.14 ± 0.02	13.1 ± 0.6
	2/1.5/1	151.8 ± 0.4	0.16 ± 0.03	11.1 ± 0.8
	2/1/1	139.8 ± 0.5	0.16 ± 0.03	13.9 ± 0.6
	1/1/1	141.7 ± 1.3	0.13 ± 0.04	10.0 ± 0.95
	1.5/1/1	160.0 ± 1.8	0.28 ± 0.03	14.2 ± 0.6
	1.5/1.5/1	144.0 ± 3.9	0.14 ± 0.02	13.1 ± 0.6
	2/1.5/1	151.8 ± 0.4	0.16 ± 0.03	11.1 ± 0.8
GC-ss-PEI	2.5/1	153.4 ± 2.2	0.19 ± 0.01	13.1 ± 0.8
	3/1	150.9 ± 2.5	0.2 ± 0.02	11.2 ± 0.8
PEI	2/1	107.5 ± 4.4	0.16 ± 0.01	22.9 ± 2.0

## Data Availability

Not applicable.

## Supplementary Materials

The following supporting information can be downloaded at: https://www.mdpi.com/article/10.3390/pharmaceutics14020373/s1. Figure S1. Polymer characterization. (A) GPC spectrum of PEI before and after thiolation; (B) GPC spectrum of GCS before and after depolymerization; and (C) 1HNMR spectrum of GCS-PDP. Figure S2. Size distribution of GNPs before and after the lyophilization. Figure S3. DNA release from GNPs, PEI/DNA, and PEI-SH/DNA in response to DTT and heparin. Figure S4. 1HNMR spectra of PEI, GCS-PDP, and GCS-ss-PEI. The arrows point to the characteristic proton resonance peaks corresponding to PEI, GCS-PDP, and GCS-ss-PEI. Figure S5. Cytotoxicity of different formulations in the medium containing 20% FBS as determined by the MTT assay. Figure S6. NSG mice without PC3 cancer cell injections were treated with GNP (1/2/1) and in vivo JetPEI containing pSur-fLuc for 3 days, and the whole-body luciferase expression was quantified.
