# Supplementary material for: A Unique Core–Shell Structured, Glycol Chitosan-Based Nanoparticle Achieves Cancer-Selective Gene Delivery with Reduced Off-Target Effects
Source: Pharmaceutics. 2022 Feb 7;14(2):373. doi: 10.3390/pharmaceutics14020373 (PMC8878887; doi:10.3390/pharmaceutics14020373)
Supplement: Supplementary file 1 [file pharmaceutics-14-00373-s001.zip › pharmaceutics-1581909-supplementary.pdf]

pharmaceutics

MDPI

Supplementary Materials: A Unique Core–Shell Structured, Glycol Chitosan-Based Nanoparticle Achieves Cancer-Selective Gene Delivery with Reduced Off-Target Effects

Bei Cheng, Hye-Hyun Ahn, Hwanhee Nam, Zirui Jiang, Feng J. Gao, Il Minn and Martin G. Pomper

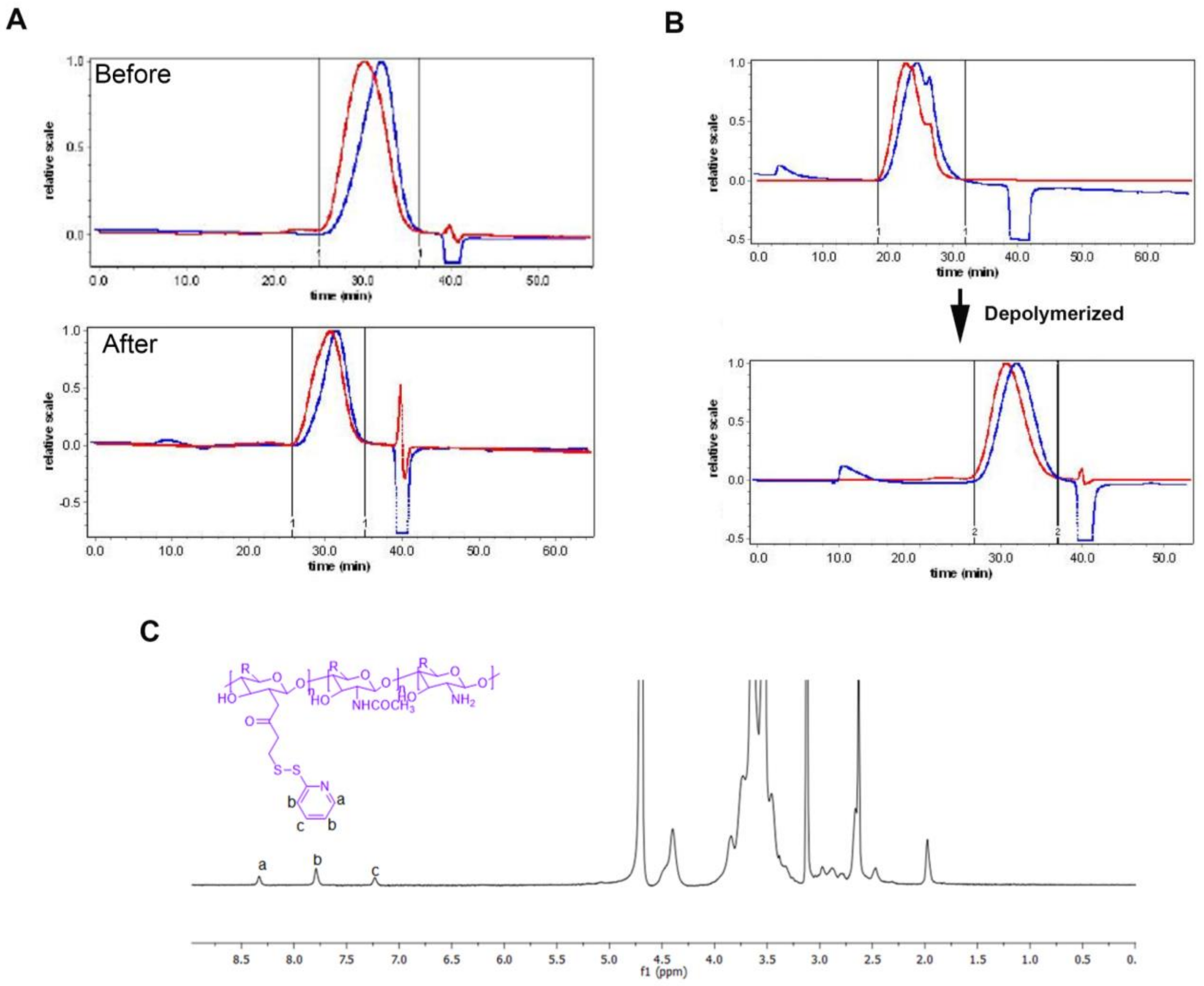

Figure S1. Polymer characterization. (**A**) Gel permeation chromatography (GPC) spectrum of PEI before and after thiolation; (**B**) GPC spectrum of GCS before and after depolymerization; (**C**) ^{1}HNMR spectrum of GCS-PDP.

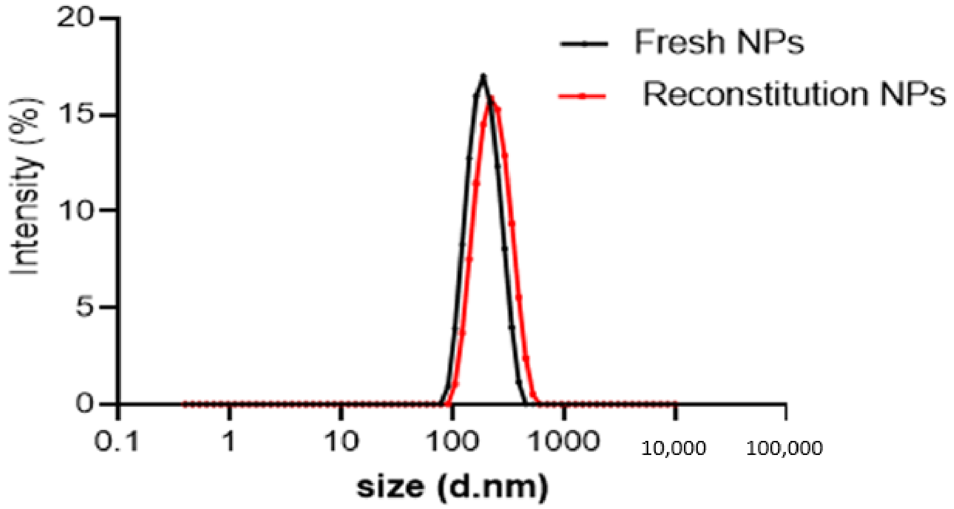

Figure S2. Size distribution of GCS-PDP/PEI-SH/DNA nanoparticles (GNP) before and after the lyophilization.

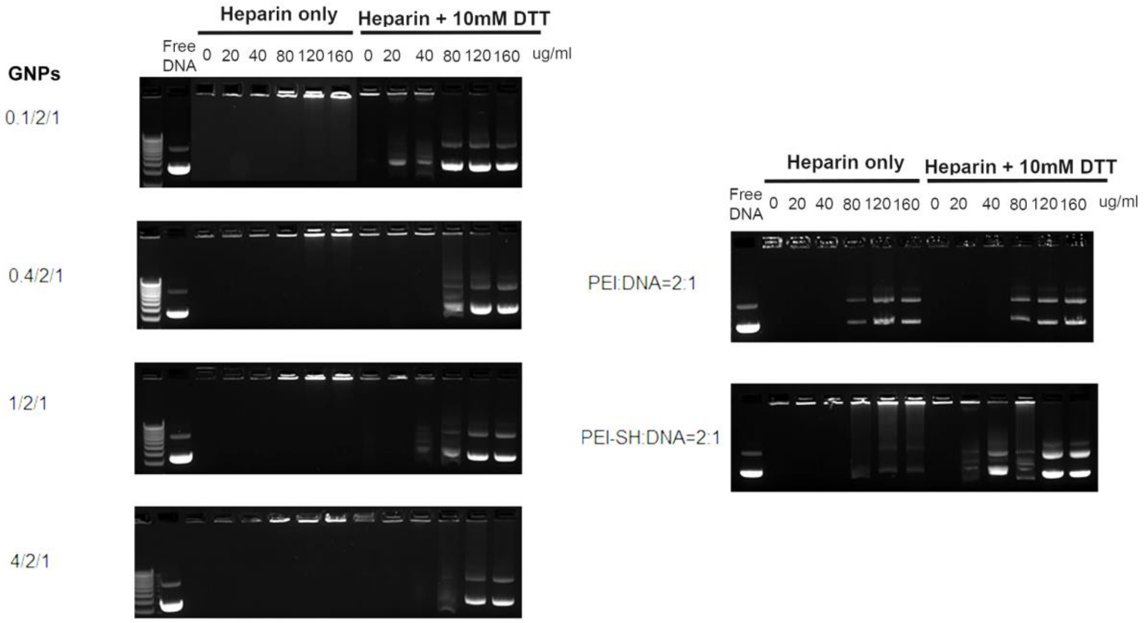

Figure S3. DNA release from GNPs, PEI/DNA, and PEI-SH/DNA in response to DTT and heparin.

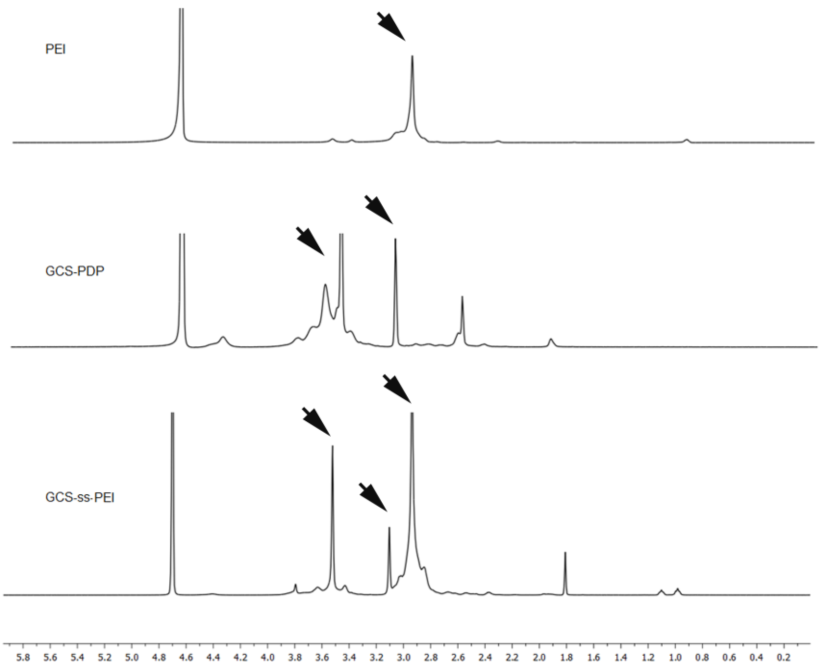

Figure S4. ^{1}HNMR spectra of PEI, GCS-PDP and GCS-ss-PEI. Arrows points to the characteristic proton resonance peaks correspond to PEI, GCS-PDP, and GCS-ss-PEI.

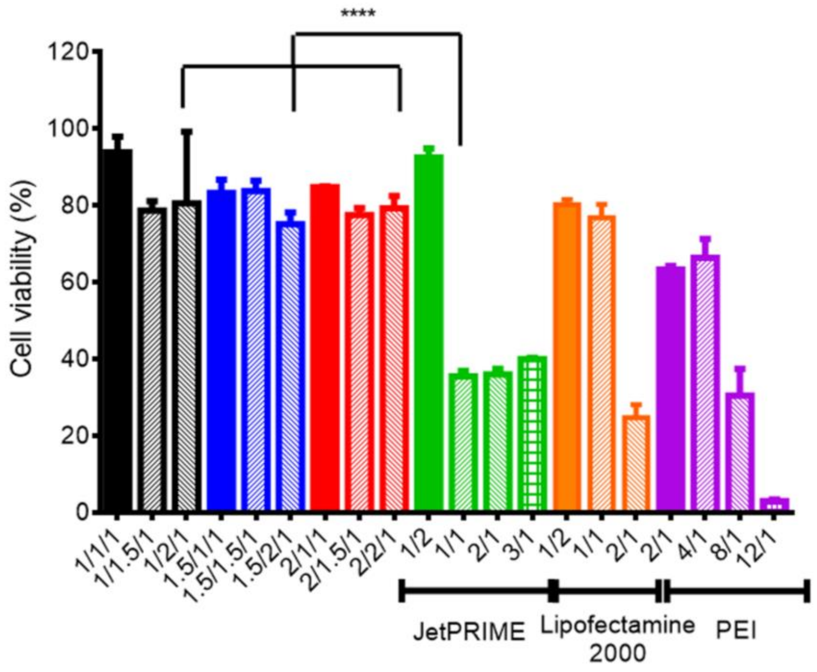

Figure S5. Cytotoxicity of different formulations in the medium containing 20% FBS as determined by MTT assay (**** $p < 0.0001$).

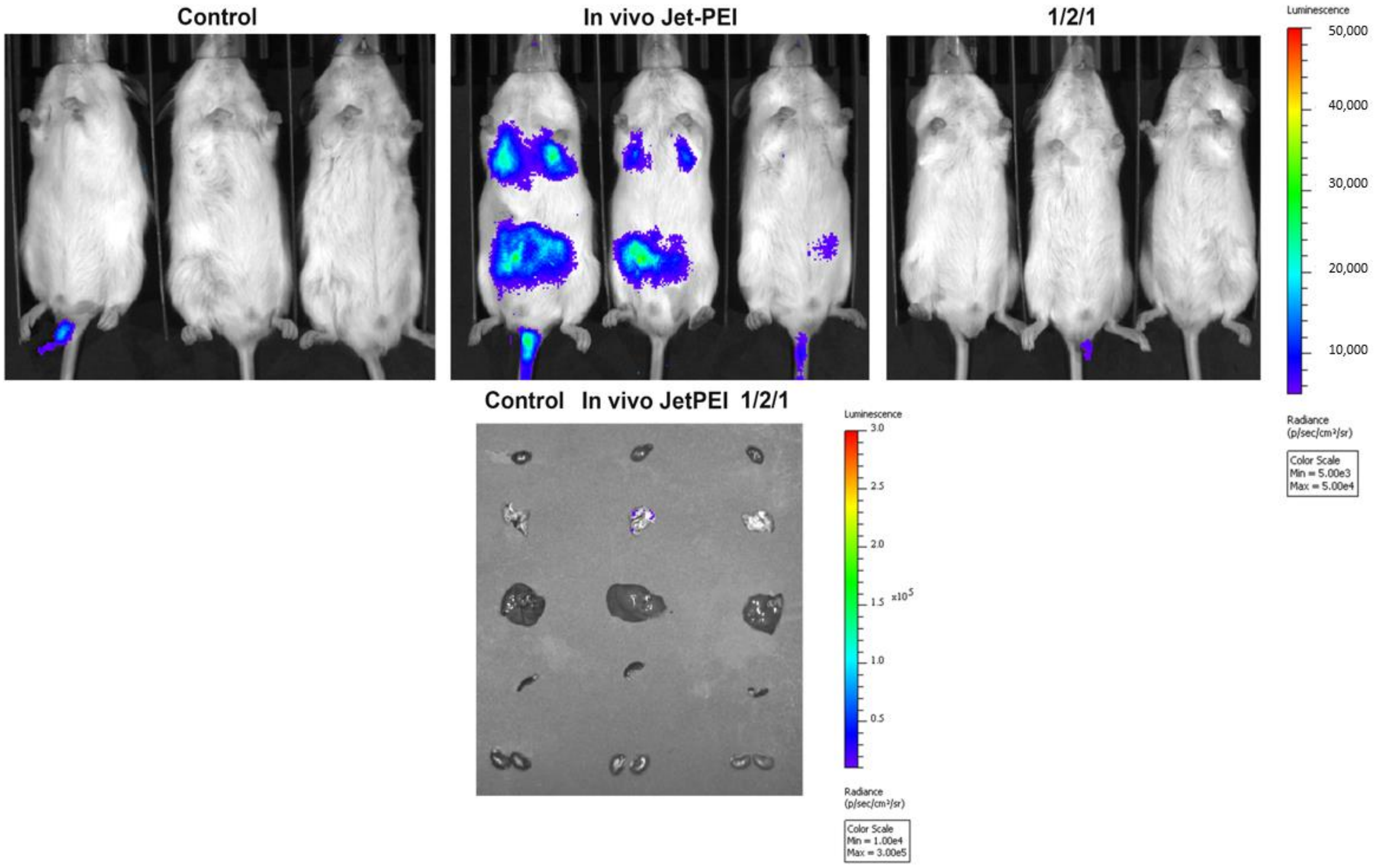

Figure S6. NSG mice without PC3 cancer cell injections were treated with GNP (1/2/1) and in vivo JetPEI containing pSur-fLuc for 3 days, and whole-body luciferase expression was quantified.
